# The small molecule SI113 synergizes with mitotic spindle poisons in arresting the growth of human glioblastoma multiforme

**DOI:** 10.18632/oncotarget.22500

**Published:** 2017-11-18

**Authors:** Claudia Abbruzzese, Giada Catalogna, Enzo Gallo, Simona di Martino, Anna M. Mileo, Mariantonia Carosi, Vincenzo Dattilo, Silvia Schenone, Francesca Musumeci, Patrizia Lavia, Nicola Perrotti, Rosario Amato, Marco G. Paggi

**Affiliations:** ^1^ Department of Research, Advanced Diagnostics and Technological Innovation, Unit of Cellular Networks and Therapeutic Targets, “Regina Elena” National Cancer Institute, IRCCS, Rome, Italy; ^2^ Department of “Scienze della Salute”, University “Magna Graecia” of Catanzaro, Catanzaro, Italy; ^3^ Department of Research, Advanced Diagnostics and Technological Innovation, Unit of Pathology, “Regina Elena” National Cancer Institute, IRCCS, Rome, Italy; ^4^ Department of Research, Advanced Diagnostics and Technological Innovation, Unit of Tumor Immunology and Immunotherapy, “Regina Elena” National Cancer Institute, IRCCS, Rome, Italy; ^5^ Department of Pharmacy, University of Genova, Genova, Italy; ^6^ Institute of Molecular Biology and Pathology (IBPM), National Research Council of Italy (CNR), c/o University “La Sapienza”, Rome, Italy

**Keywords:** glioblastoma multiforme, SI113, SGK1, vincristine, in vivo cancer therapy

## Abstract

Glioblastoma multiforme (GBM) is the deadliest brain tumor. State-of-art GBM therapy often fails to ensure control of a disease characterized by high frequency of recurrences and progression. In search for novel therapeutic approaches, we assayed the effect of compounds from a cancer drug library on the ADF GBM cell line, establishing their elevated sensitivity to mitotic spindle poisons. Our previous work showed that the effectiveness of the spindle poison paclitaxel in inhibiting cancer cell growth was dependent on the expression of RANBP1, a regulatory target of the serine/threonine kinase SGK1. Recently, we developed the small molecule SI113 to inhibit SGK1 activity. Therefore, we explored the outcome of the association between SI113 and selected spindle poisons, finding that these drugs generated a synergistic cytotoxic effect in GBM cells, drastically reducing their viability and clonogenic capabilities *in vitro*, as well as inhibiting tumor growth *in vivo*. We also defined the molecular bases of such a synergistic effect.

Because SI113 displays low systemic toxicity, yet strong activity in potentiating the effect of radiotherapy in GBM cells, we believe that this drug could be a strong candidate for clinical trials, with the aim to add it to the current GBM therapeutic approaches.

## INTRODUCTION

Glioblastoma Multiforme (GBM), the most lethal primary brain tumor, presents unique challenges to therapy due to its location, aggressive biological behavior and diffuse infiltrative growth, resulting in disproportionately high morbidity and mortality. High-tech surgery, molecular characterization [1], as well as the use of novel targeted therapies did not change substantially GBM patients’ prognosis, which remains definitely dismal [2, 3].

Due to the compelling need for novel and effective therapeutic approaches toward GBM, we recently assayed a 349-compound library of anticancer agents using the ADF GBM cells [4] *in vitro*, determining the strong efficacy of mitotic spindle poisons in restraining their growth. Our group had already shown that the effectiveness of paclitaxel, a drug belonging to the same class of antimitotic drugs, is strictly related to the expression of the RAN-binding protein 1 (RANBP1), a major RAN GTPase effector. RANBP1, in turn, is regulated by the serum- and glucocorticoid-regulated kinase 1 (SGK1), a serine/threonine kinase that plays a key role in cancer signal transduction [5–9].

In GBM, the PI3K/mTOR signaling pathway, involving both AKT and SGK1, appears frequently dysregulated [1]. Recently, a small molecule inhibitor, SI113, was demonstrated to inhibit selectively the kinase activity of SGK1 *in vitro* [10]. In addition, SI113 has proved valuable, alone or in combination, in restraining *in vitro* and *in vivo* hepatocarcinoma cell growth, with no apparent short-term side effects in mice [11, 12]. We previously assayed SI113 on three established GBM cell lines, where this drug was able to impact on the effect of radiotherapy and oxidative stress, also inducing in these cells an autophagic cell death [13].

With this background, we decided to explore the outcome of the association between SI113 and selected mitotic spindle poisons on GBM cell viability. We report that these drug associations produce indeed a synergistic cytotoxic effect, *in vitro* and *in vivo*.

## RESULTS

### Screening of a drug library

A 349-compound library was screened by assaying the effect of each single drug on the ADF cells [4] survival *in vitro*. Cells were incubated for 48 h in the absence or presence of any of the library compounds, which were tested at a concentration of 10 nM. Drug effect was then evaluated using a cell viability assay. Thirteen out of 349 drugs were able to decrease cell viability below 20% at a 48 h exposure time (Supplementary Figure 1). Among the effective drugs, we found the microtubule-destabilizing agent Vincristine (VCR), a naturally occurring Vinca alkaloid [14, 15], the microtubule-stabilizing agents Epothilone A (EPO-A) and Epothilone B (EPO-B) [16] and also Ispinesib (ISP), a highly specific inhibitor of the kinesin spindle protein KSP1/Eg5 [17]. As expected, taxanes also resulted quite effective in reducing ADF cell viability (see below). All mentioned drugs, albeit acting in specific pathways, belong to the large class of antimitotic drugs and ultimately share a common target process, i.e. mitotic spindle assembly, thus hampering mitotic progression at prophase/prometaphase and preventing the generation of two viable daughter cells [15, 18, 19].

### Mitotic spindle poisons synergize with SI113 in restraining the growth of GBM cell lines: Cell viability assay

**Figure 1:**
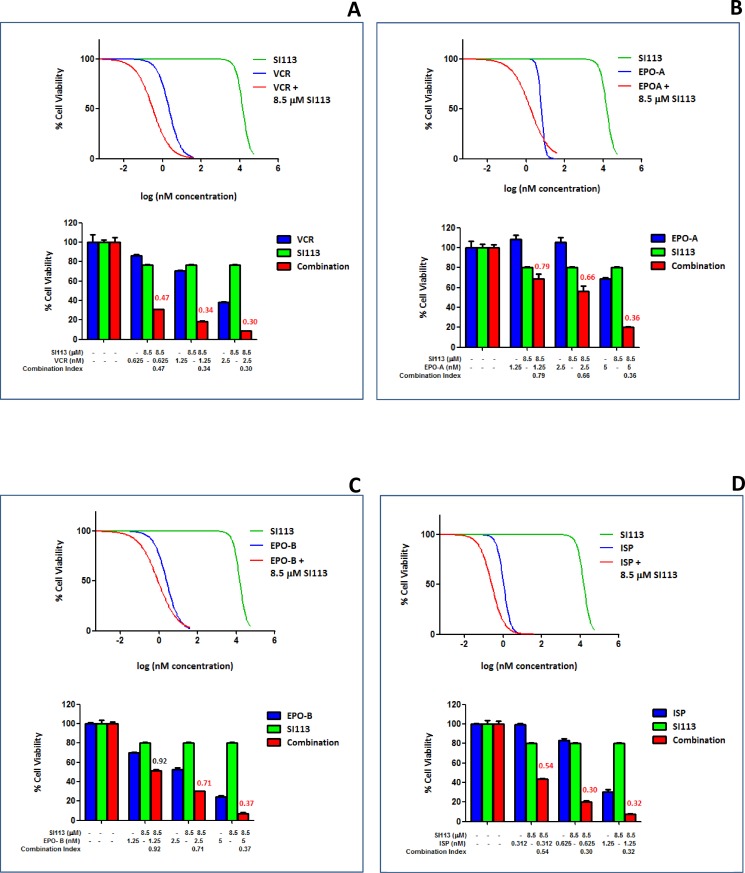
Synergistic effect of SI113 and VCR, EPO-A, EPO-B or ISP in restraining the growth of the ADF cells: Cell viability assay (**A**) Dose-response curves showing the effect of SI113 (green), VCR (blue) and VCR plus a constant 8.5 µM SI113 concentration (red) on percent viability of ADF cells. Histograms show ADF cell viability at selected drug concentrations, as indicated, to highlight the effect of the association of the two drugs. Synergy is characterized by a Combination Index <0.8 and, when present, its value is reported in red. (**B**) As in panel A, except for the use of EPO-A as the companion drug. (**C**) As in panel A, except for the use of EPO-B as the companion drug. (**D**) As in panel A, except for the use of ISP as the companion drug. Control values were generated by adding the maximum amount of solvent(s) to the cells.

RAN and RANBP1 modulate the intrinsic spindle stability [20, 21]. In previous work, we showed that cancer cells displaying increased SGK1 activity, and hence increased RANBP1 levels, become less sensitive to the effects of the microtubule-stabilizing agent paclitaxel [22]. Consistent with this, SGK1 down-regulation sensitizes cancer cells to this drug [6, 23]. Further data also point out the effect of the SGK1 kinase inhibitor SI113 in restraining *in vitro* and *in vivo* proliferation in several cancer cells [10, 11], including GBM cells [13]. We thus evaluated the effect of the treatment with SI113, VCR, EPO-A, EPO-B and ISP on three GBM cell lines, i.e. ADF, U373MG and T98G, in a cell viability assay. Titration of the effectiveness of these drugs determined their half-maximal inhibitory concentration (IC50) value, assayed by measuring the survival of the GBM cell lines after 48 h of exposure (Table 1). Next, in order to highlight potential synergistic effects, SI113 was employed on the ADF cells at a concentration of 8.5 µM, corresponding to its IC20, i.e. the concentration able to reduce cell viability of 20%, thus allowing an 80% survival. We tested this fixed concentration for SI113 in association with increasing doses of VCR, EPO-A, EPO-B or ISP, and assessed cell viability after 48 h. Using the algorithm described by Fransson *et al.* [24], we analyzed the outcome of the combinations SI113/VCR, SI113/EPO-A, SI113/EPO-B or SI113/ISP compared with that of each of the compounds used as single agents. Indeed, in the presence of SI113 at its IC20 dose, titration of VCR, EPO-A, EPO-B and ISP yielded a decrease in cell viability which was definitely attributable to a synergistic effect within an extended range of concentrations for all four companion drugs (Figure 1A–1D).

**Table 1:** IC50 values for SI113, VCR, EPO-A, EPO-B and ISP in ADF, U373MG and T98G human GBM cell lines

Cell lines	IC50 (nM)				
	SI113	VCR	EPO-A	EPO-B	ISP
**ADF**	14482	2.01	4.53	2.12	1.81
**U373MG**	12773	2.68	16.96	6.00	12.02
**T98G**	6601	6.44	5.34	2.17	4.76

Values were determined by titrating the effect of each drug, by means of a cell viability assay, after 48 h of treatment. The data reported are the average of two different experiments performed in triplicate.

### SI113 induces apoptosis and autophagic cell death in the ADF cells

**Figure 2:**
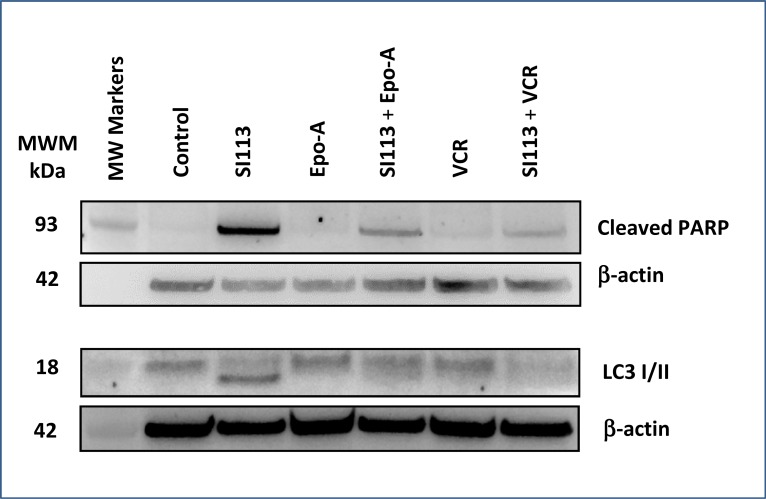
SI113 induces apoptosis and autophagic cell death in the ADF cell line ADF cells were incubated for 48 h in the absence or presence of SI113 (8.5 µM), EPO-A (3.5 nM), VCR (0.625 nM) or the combination of SI113 and EPO-A or SI113 and VCR. After cell lysis, SDS-PAGE and protein transfer on a PVDF membrane, cleaved PARP and LC3 were determined by Western blot. Control values were generated by adding the same amount of solvent(s) to the cells. β-actin determination was performed as loading control.

In the experimental setting described above, S113-dependent apoptosis was highlighted by an increased expression of cleaved PARP detected by Western blot. In particular, cleaved PARP, a cell death marker typically linked to DNA damage in cycling cells, was clearly detectable when SI113 was used alone, but was induced by neither EPO-A nor VCR, both of which arrest the cell cycle in prophase/prometaphase, thus inducing mitotic cell death. When cells were co-treated with the combinations SI113 plus EPO-A or SI113 plus VCR, PARP cleaved form could be appreciated, albeit less intensely than with SI113 alone, consistent with the cell cycle inhibitory effect exerted by EPO-A and VCR in G2-M. In parallel, SI113-dependent triggering of the autophagic process was clearly reflected by the shift in the LC3 apparent molecular mass. Autophagy was not evident when cells were exposed to either EPO-A or VCR alone, whereas both drugs yielded the co-appearance of higher and lower molecular mass LC3 forms when used in combination with SI113 (Figure 2). The original blots that have been cropped to obtain the images in Figure 2 are shown in Supplementary Figure 6.

### Spindle poisons, but not SI113, induce mitotic aberrations in ADF cells

**Figure 3:**
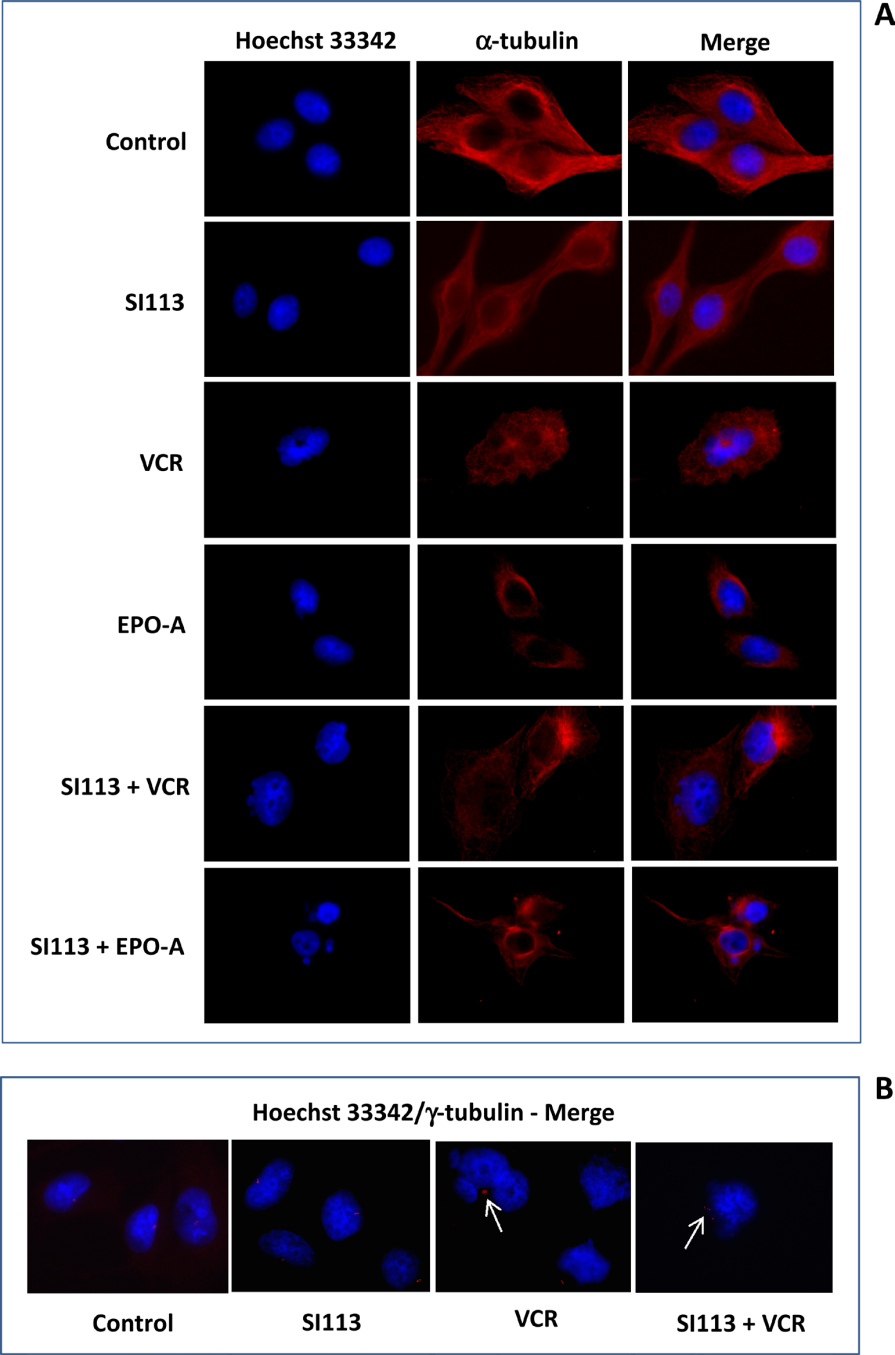
Spindle poisons induce mitotic aberrations in ADF cells (**A**) ADF cells were incubated for 48 h in the absence or presence of 8.5 µM SI113, 0.625 nM VCR, 3.5 nM EPO-A or the combination of SI113 and VCR or SI113 and EPO-A. Cells were then stained with Hoechst 33342 to highlight nuclei (blue, left column) and α-tubulin (fluorescent antibody, red, central column). Merging is shown in the right column. Cells incubated in the presence of VCR or EPO-A, regardless the presence or absence of SI113, appear larger, with microtubule destabilization and hyperconsensed chromatin, harboring multiple nuclei, micronuclei and/or multi-lobulated nuclei. (**B**) ADF cells stained with Hoechst 33342 and γ-tubulin (merged). VCR- or SI113 plus VCR- cells, treated as above, showed condensed chromatin (M-arrested), displaying large γ-tubulin foci (arrows), suggestive of overduplicated, clustered centrosomes. In both panels, control values were generated by adding the same amount of solvent(s) to the cells.

Spindle poisons act by affecting microtubule functions, thus arresting mitosis and inducing functional perturbations culminating in mitotic cell death [25–27]. ADF cells were exposed to SI113, VCR and EPO-A or to the combination of SI113 with either mitotic spindle poisons as above; then fluorescence microscopy analysis for nuclei (Hoechst 33342, blue, left column) and α-tubulin (fluorescent antibody, red, central column) was done. Merged images are shown in the right column (Figure 3A). Image analysis showed large cells with microtubule destabilization and hyperconsensed chromatin, harboring multiple nuclei, micronuclei and/or multi-lobulated nuclei, all features reflecting failed chromosome segregation, or mis-segregation of one or few chromosomes (giving rise to micronuclei), favoring the onset of mitotic cell death, when cells were exposed to VCR or EPO-A, with our without SI113.

In parallel assays, cells were stained for nuclei (Hoechst 33342) and γ-tubulin (fluorescent antibody, red) for centrosome detection. Untreated (Control) cells displayed one or two centrosomes, indicating cells in G1 or G2 phase, respectively, of the cell cycle. Most of SI113-treated cells displayed two centrosomes, indicating cells arrested in G2. Cells treated with VCR showed condensed chromatin (M-arrested), displaying often large γ-tubulin foci (arrow), suggestive of overduplicated, clustered centrosomes. Overduplicated centrosomes were also evident in cells treated with both SI113 and VCR (arrow) (Figure 3B). Supplementary Figure 2 shows the complete experimental set, where ADF cells were treated as indicated and stained for nuclei (Hoechst 33342, blue, left column) and γ-tubulin (fluorescent antibody, red, central column). Merged images are shown in the right column. Of note, overduplicated and clustered centrosomes were also evident in EPO-A- and SI113/EPO-A-treated cells.

These results indicate that mitotic aberrations were elicited by mitotic spindle poisons alone, as expected, and in combination with SI113, thus the synergistic effect in causing cell toxicity was apparently not ascribable to direct interference of SI113 with the mitotic apparatus. Here, SI113 more likely represents an additional impairment for these cells due to its indirect interference with RANBP1 via SGK1 inhibition [6, 10].

### SI113 synergizes with VCR and EPO-A in restraining ADF cell proliferation - clonogenic assay

**Figure 4:**
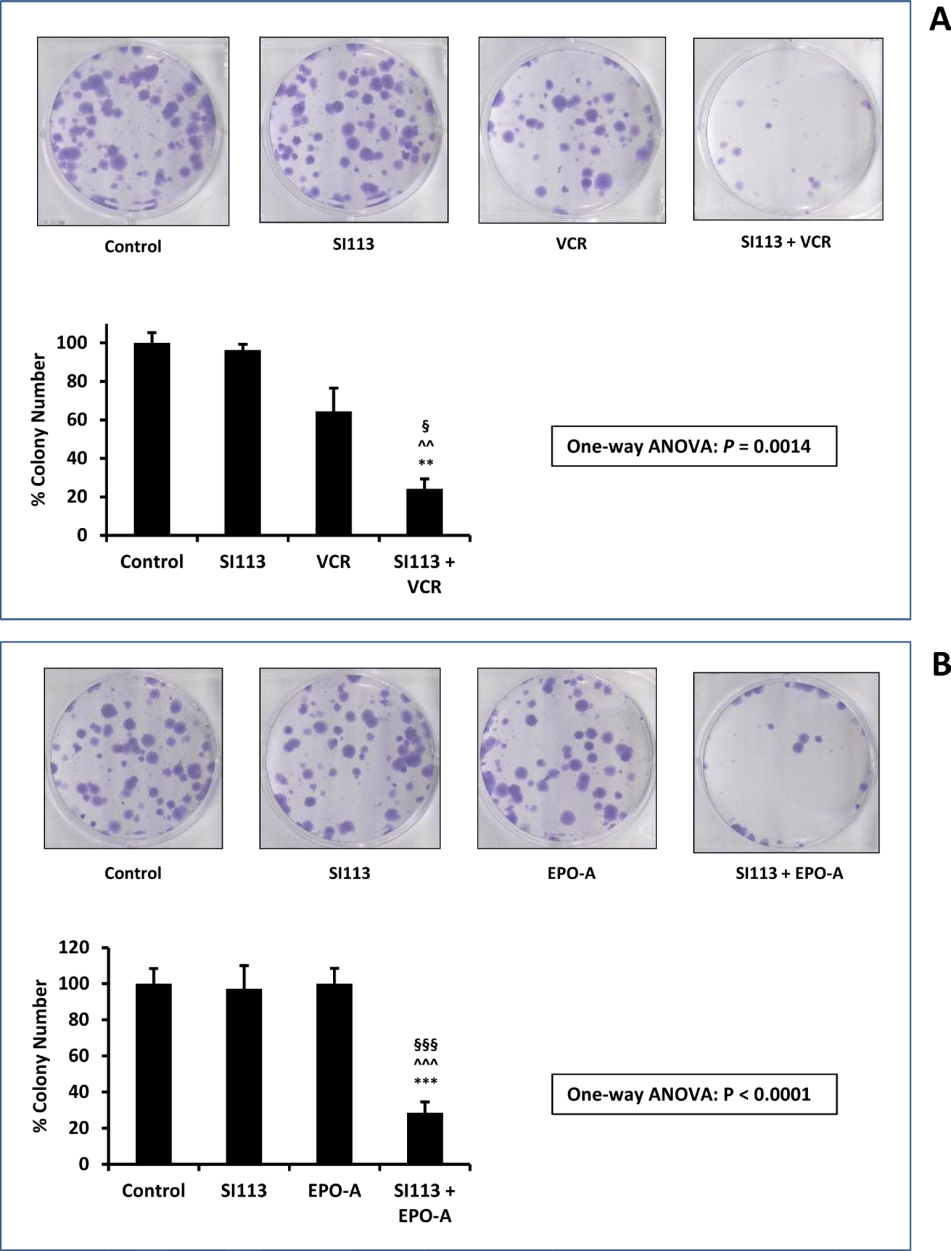
Synergistic effect of SI113 plus VCR, and SI113 plus EPO-A in restraining the growth of the ADF cells: Clonogenic Assay (**A**) ADF cells were exposed to solvent(s) (Control), 8.5 µM SI113, 0.312 nM VCR or their association for 48 h and then allowed to grow and form colonies for the subsequent 12 d. Cell colonies, after staining with crystal violet (upper panels), were counted and the values reported as percent colony number in the histogram (lower panel). Statistical significance is also indicated (^*^significance vs. Control; ^^^significance vs. SI113; ^§^significance vs. VCR). (**B**) As in panel A, except for the use of 0.5 nM EPO-A as the companion drug. Statistical significance is also indicated (^*^significance vs. Control; ^^^significance vs. SI113; ^§^significance vs. EPO-A).

At this point we wished to rely on an independent drug toxicity assessment. We therefore performed a clonogenic assay, in which ADF cells were exposed to the drug(s) or their respective solvent(s) for 48 h, washed, and allowed to grow and form colonies for the subsequent 12 d. Colonies were then visualized by crystal violet staining, and the effect of the drug(s) was evaluated via the inhibition of cell colony formation, when compared to the control. VCR and EPO-A doses were scaled down to the concentrations indicated in the Figure legend, according to the different experimental demands. Indeed, this experimental setting confirmed a highly significant decrease in cell colony formation in the presence of combined SI113 plus VCR or EPO-A compared with either drug alone. In Figure 4A and 4B, both representative images and histograms depict the scarce effect of each single treatment in reducing colony formation, with either SI113, or VCR or EPO-A, contrasting with the dramatic effect evoked by the combination of SI113 with the two mitotic spindle poisons.

### SI113 reduces the expression of stemness genes in ADF cells

**Figure 5:**
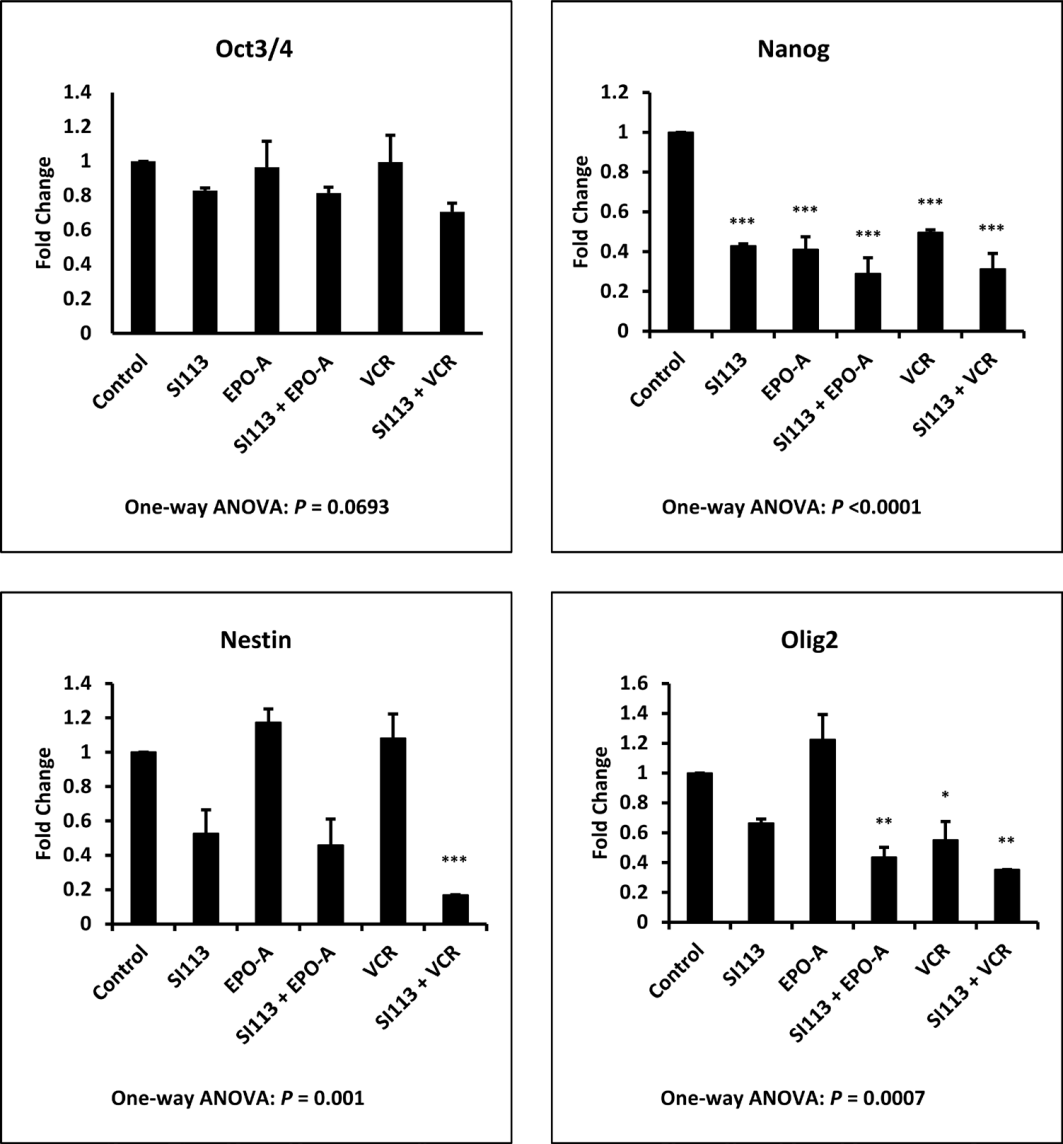
SI113 reduces the expression of stemness genes in ADF cells ADF cells were incubated for 48 h in the presence of solvent(s) (Control), 8.5 µM SI113, 3.5 nM EPO-A, 0.625 nM VCR, or a combination of them, as indicated. After nucleic acid extraction, quantitative PCR for OCT3/4, NANOG, NESTIN and OLIG2 mRNA expression was performed. Results, normalized against GAPDH expression, are represented as histograms and the statistical significance toward Control, when present, is reported on each single column.

Aiming to identify the mechanism(s) underlying the reduction in clonogenic potential observed in combination treatments, as described above, we analyzed the effect of SI113, VCR, EPO-A, as well as the combination of SI113 with both mitotic spindle poisons, on the expression of several stemness genes, most of them specific for neural cells. ADF cells were exposed to drug(s) or their respective solvent(s) for 48 h and then subjected to transcript analysis. As shown in Figure 5, treatment with SI113, alone or in combination with EPO-A or VCR, yielded a modification of mRNA expression for OCT3/4, NANOG, NESTIN and OLIG2, all GBM or glioma stemness markers [28]. Statistical significance was reached for NANOG, NESTIN and OLIG2 only when compared with Control values, as indicated by the asterisks in the Figure. Looking at the effect of SI113, EPO-A and VCR as single agents, NANOG appeared significantly down-regulated by all three drugs and OLIG2 only by VCR, alone or in combination with SI113. Conversely, NESTIN down-regulation resulted significant only when SI113 and VCR were used in combination and no significant down-regulation was observed when the values were compared with the effect of SI113 alone.

### SI113 and VCR cooperate in restraining the growth of ADF xenografts in immunocompromised mice

On the bases of the *in vitro* studies described above, we chose to employ the ADF cells in order to generate xenografts for *in vivo* experimentations. Cells (2.5 × 10^6^ cells) were implanted in the flanks of NOD/SCID female mice for *in vivo* treatment with SI113, VCR or the combination of both. Treatment of the four experimental arms, control, SI113 (5.4 mg/kg), VCR (1.1 μg/kg, a dose deliberately below those used in the clinics) or SI113 plus VCR, started when the tumor reached the volume of about 130 mm^3^. Drugs (or vehicles) were administered five days/week and tumor growth was monitored every 4 days for 20 days after the beginning of the treatment. Mice were then sacrificed when tumors in any of the four groups reached the volume of 1000 mm^3^. Tumors were then excised, weighed and formalin-fixed/paraffin-embedded (FFPE).

**Figure 6:**
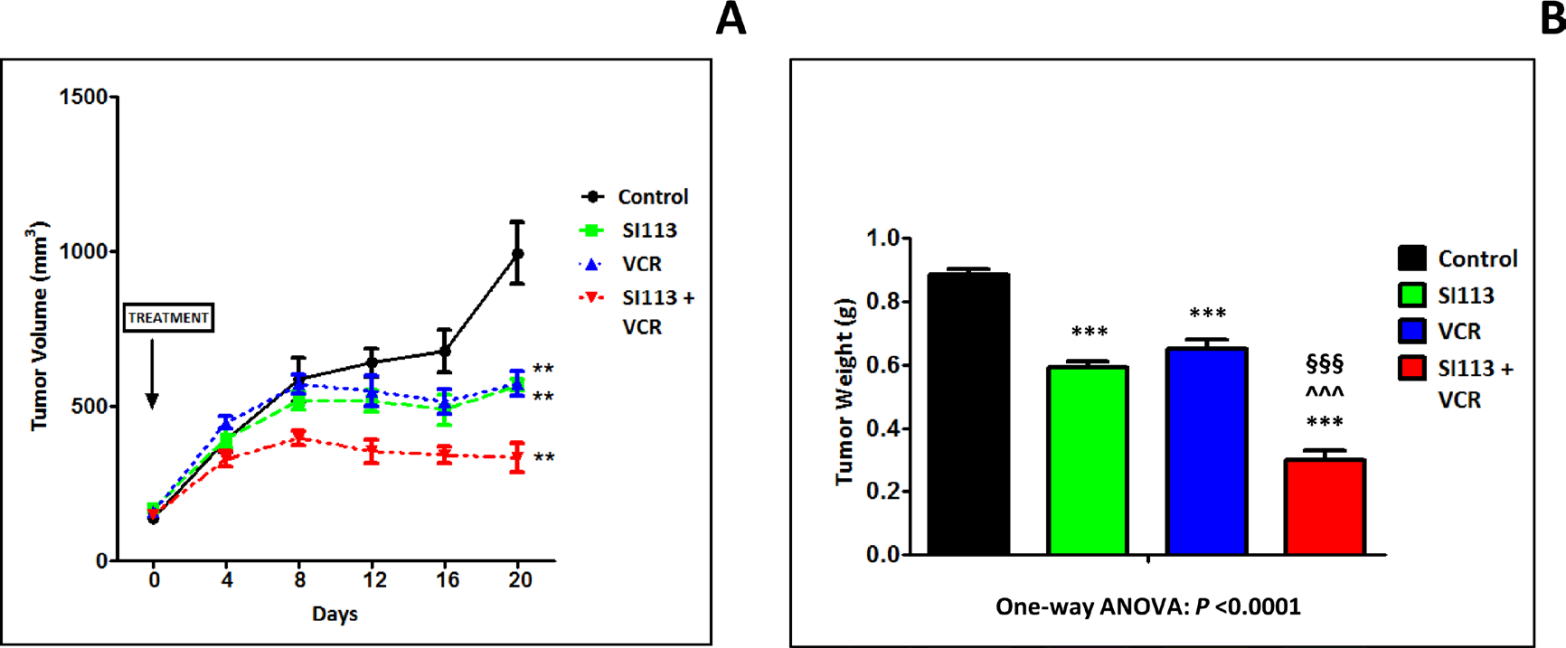
SI113 synergizes with VCR in restraining the growth of ADF xenografts in immunocompromised mice ADF cells (2.5 × 10^6^ cells) were implanted in the flanks of NOD/SCID female mice. When tumors reached the volume of about 130 mm^3^, mice were divided into four arms, i.e. Control, SI113, VCR or SI113 plus VCR for the *in vivo* treatment. Drugs (or their solvents in the Control arm) were administered five days/week, and tumor growth was monitored every 4 days for 20 days after the beginning of the treatment. (**A**) The graph shows the curves for tumor growth for control and animals treated with SI113, VCR or their association. Statistical analysis was done using the Student’s two-tailed *t* test (^**^ = *P* < 0.01). Data are expressed in mm^3^ ± Standard Error (SE). (**B**) At day 20 from the beginning of the treatment, mice were sacrificed and tumors were excised and weighed. The histogram represents tumor weight for each experimental arm expressed in g ± SE. In this panel, statistical analysis among groups was done using the One-way ANOVA test followed by the Tukey’s Multiple Comparison Test. Statistical significance is also indicated (^*^significance vs. Control; ^^^significance vs. SI113; ^§^significance vs. VCR).

Under these experimental conditions, a remarkable reduction in tumor volume was apparent for the SI113 plus VCR arm since day 8 of treatment. Eventually, at day 20, both tumor volume (Figure 6A) and tumor weight (Figure 6B) measurements clearly indicated a statistically significant delay in tumor growth in SI113-, VCR- and SI113 plus VCR-treated arms. Of note, the combo treatment arm resulted by far more effective than any of the single drug-treated arms.

### Effect of SI113, VCR, EPO-A and SI113/VCR or SI113/EPO-A association on U373MG and T98G GBM cells

Aiming to assay the effect of SI113, VCR and EPO-A on other cell models, we replicated selected experiments using the U373MG [29] and T98G [30] GBM cell lines.

When cell viability assay was performed using the U373MG cells, synergy was found when SI113 (used at 7.9 µM concentration, i.e. the IC20 of the drug for this cell line) was administered together with increasing VCR doses (Supplementary Figure 3A). Instead, co-administration of SI113 with increasing EPO-A doses brought to a merely additive effect (Supplementary Figure 3B). Similar results were obtained when the T98G GBM cell line was employed using SI113 (used at 5.5 µM concentration, i.e. the IC20 of the drug for this cell line) plus increasing concentrations of VCR or EPO-A (Supplementary Figure 3C and 3D).

Clonogenic assays were then performed using the U373MG cells. A significant inhibition in colony formation was found when SI113 was administered together with VCR, whereas co-administration of SI113 with EPO-A brought to a less marked, yet still statistically significant, effect (Supplementary Figure 4A and 4B). When T98G cells were employed using SI113, VCR produced a significant reduction of colony formation; a similar result was observed for the association between SI113 and EPO-A (Supplementary Figure 5A and 5B).

## DISCUSSION

Despite optimal treatment according to Stupp *et al.* [31], the median survival of GBM patients is only 12 to 15 months, and less than 5% of patients survive for more than 5 years after diagnosis [1, 3].

In search for novel therapeutic approaches, we recently showed that the small molecule SI113 is active in negative modulation of major survival processes in GBM cells [13]. Here, by means of a drug library screening, we found that the ADF cell line was quite sensitive to mitotic spindle poisons, also showing that their association with SI113 elicited a synergistic effect in restraining GBM cell growth and clonogenic capability. Indeed, we report that direct disruption of the mitotic apparatus by VCR or EPO-A and, concomitantly, impairment of RANBP1 function via SI113-dependent SGK1 inhibition [10], heavily restrained GBM cell growth *in vitro* and *in vivo*. In addition, the known effect of SI113 on the PI3K/mTOR signal transduction pathway - SI113 being an inhibitor of the SGK1 kinase activity [10] - may play a further role in impeding GBM cell growth and survival. The reason underlying the noticeable *in vitro* and *in vivo* GBM cell impairment described in our results can be thus attributed to the combination of these effects.

A key point in GBM patients’ treatment is the drug permeability through the Blood-Brain Barrier (BBB), which may represent an obstacle to drug distribution in the CNS structures. Usually, BBB appears damaged in high-grade gliomas, due to the disruption of the normal cerebral vascular architecture and to tumor-induced neo-angiogenesis. While SI113 is predicted to pass the BBB [13], concerns are raised for some spindle-targeting drugs, e.g. VCR and paclitaxel [15], whereas epothilones are demonstrated to pass the BBB [15, 32]. Nevertheless, in spite of its poor permeability through an intact BBB, VCR along with procarbazine and lomustine (CCNU), i.e. the PCV regimen, is employed in some CNS tumors [33]. In our *in vivo* studies we have coupled SI113 with VCR, also considering that the latter belongs to the WHO Model List of Essential Medicines (http://www.who.int/medicines/publications/essentialmedicines/en/), being considered an effective and widely employed drug. In addition, VCR treatment has recently been reported to trigger an immune response [34]. Considering that a reduced VCR penetration in normal brain could be circumvented in several effective ways [35], our results represent a proof-of-concept regarding the synergy between SI113 and VCR in impeding cancer growth.

On the whole, the results reported here outline a clear-cut effect of SI113, alone or in association with mitotic spindle poisons, on GBM vital processes by inducing apoptosis and autophagic cell death. The effects of the mitotic spindle poisons appear instead confined to an induction of cell death by mitotic arrest. Thus, SI113 synergizes with VCR in potently inhibiting proliferation of ADF cells *in vitro* and *in vivo* and also reducing cell clonogenicity. Specifically, the effects of these drugs, as described here, are of potential relevance when envisaging novel combination therapeutic strategies.

State-of-art first line treatment of newly diagnosed GBM patients [31] (maximal surgical resection followed by radiotherapy and chemotherapy with temozolomide) produces only minor benefits on patients’ survival time. Recent studies suggest that distinct molecular subtypes exist within the broader pathologic diagnosis of GBM, and strengthen the notion that responses to specific treatments vary based on the molecular subtype [36, 37]. The intrinsic GBM heterogeneity [38, 39] remains a real obstacle for complete eradication of this disease, where tumor recurrence arises in the majority of the patients and no standard of care is presently established for recurrent or progressive GBM. In this scenario, disease relapses driven by cells resistant toward previous - also precision medicine-driven - approaches is almost the rule. This could possibly be faced using therapeutic strategies designed to hit general cancer cell survival pathways, as both SI113 and mitotic spindle poisons do. The use of these drugs might be of great help also in providing a more incisive first-line treatment, when associated with the GBM standard therapeutic approach.

Finally, taking into account that SI113 potentiates the effects of radiotherapy in GBM cells [13], this drug can be considered as a strong candidate for GBM therapy in a clinical setting. Thus, we feel that all these elements converge to support the idea of a Phase 1 clinical trial for SI113 safety and dosage assessments.

## MATERIALS AND METHODS

### GBM cell lines

ADF human GBM cells [4] were a gift from Dr. W. Malorni (Istituto Superiore di Sanità, Rome, Italy). U373MG and T98G GBM cells were provided by Dr. C. Leonetti (“Regina Elena” National Cancer Institute, Rome, Italy). All cells were Mycoplasma-free and were used for a maximum of 20 passages after thawing. Cells were cultured at 37°C, in a humidified atmosphere of 5% CO_2_ and 95% air, in DMEM (Life Technologies, Inc., Grand Island, NY, USA), supplemented with 10% fetal bovine serum plus 1% penicillin-streptomycin.

### Anti-Cancer-Compound Library, SI113 and Cell Viability

We used the “Anti-cancer Compound Library” (L3000, Selleckchem Inc., Houston, TX, USA) consisting of 349 compounds all in use or under clinical trial. After the library screenings, VCR was purchased from Pfizer Inc. (New York, NY, USA) as a 1 mg/ml solution, while EPO-A was purchased from Sigma-Aldrich, St. Louis, MO, USA and dissolved in DMSO at a 100 μM concentration. Data for each single compound were reported as percent inhibition toward solvent-treated cells (100%).

SI113 was synthesized as previously reported [40]. The drug was diluted in DMSO at a 50 mM concentration.

### Cell viability assays

For drug library screening and toxicity assays, cells were seeded in 96-well plates at 5 × 10^3^ cells/well and successively exposed to the drugs (or their solvent) for 48 h. The assay was performed by means of the CellTiter-Glo Luminescent Cell Viability Assay (Promega KK, Tokyo, Japan), employing all the 349 compounds at the concentrations of 10 nM. For selected drugs (VCR, EPO-A, EPO-B, ISP and SI113), a dose-response curve was accomplished for IC50 evaluation. Cell viability was determined by means of a GLOMAX 96 Microplate Luminometer (Promega).

### Colony-forming assays

Cells were plated at 100–200 cells/well in 6-well plates. The following day vehicle, SI113, VCR, EPO-A or a combination of two compounds (as indicated) was added and the culture was incubated for 48 h. After washing, cells were incubated for 12 d and then stained with a 5% crystal violet solution in order to count the colony number.

### Immunoblot analysis

Cells were treated as described [6] and probed with anti-cleaved PARP (Asp214) PAb (1:500, Cell Signaling Technology, Inc. Danvers, MA, USA); anti-LC3 PAb (1:400, MBL International, Woburn, MA, USA); anti-β-actin MAb (1:10000, MP Biomedicals, Aurora, OH, USA).

### Immunofluorescence

ADF cells were plated in 6-well plates at a density of 1 × 10^4^ cells per well on glass cover slips. After the treatments, cells were fixed in a 4% paraformaldehyde solution and permeabilized with 0.1% Triton-X100 in PBS at room temperature. After blocking in 1% BSA in PBS, cells were incubated with α-tubulin monoclonal antibody (1:100, Calbiochem, San Diego, CA, USA) and then with an Alexa 594-conjugated secondary antibody (Life Technologies). Another set of ADF cells, after treatments, was fixed in ice-cold methanol, blocked and immunostained for mitotic center with a γ-tubulin monoclonal antibody (Santa Cruz Biotechnology) at a 1:200 dilution. Nuclear counterstaining was performed by means of Hoechst 33342. After extensive washing, immunostaining was visualized using an Olympus BX53 Fluorescence Microscope (Olympus Corporation of the Americas, Center Valley, PA, USA) equipped with a Jenoptic ProgRes MF (Jena, Germany) digital camera. Images were then processed using the NIS-Elements HC 4.2 software (Nikon Instruments, Campo Bisenzio, Florence, Italy).

### Real-time PCR

Total RNA was extracted from ADF cells using the MasterPure RNA Purification Kit (Epicentre Biotechnologies, Madison, WI, USA). RNA was reverse-transcribed into cDNA using the High Capacity cDNA Reverse Transcription Kit (Applied Biosystems Inc., Foster City, CA, USA) and subject to 7500 Fast System Real Time PCR (Bio-Rad Laboratories, Hercules, CA, USA) with PowerUp SYBR Green Master Mix (Applied Biosystems). Primers were designed for different targets as specified below; primers designed for GAPDH were applied as internal controls:

OCT3/4 [41] Sense: 5′-TGGAGAAGGAGAAGCTGGAGCAAAA-3′

Antisense: 5′-GGCAGATGGTCGTTTGGCTGAATA-3′

NANOG [42] Sense: 5′-CAAAGGCAAACAACCCACTT-3′

Antisense: 5′-ATTGTTCCAGGTCTGGTTGC-3′

NESTIN [43] Sense: 5′-CAGCGTTGGAACAGAGGTTGG-3′

Antisense: 5′-TGGCACAGGTGTCTCAAGGGTAG-3′

OLIG2 [44] Sense: 5′-CCAGAGCCCGATGACCTTTTT-3′

Antisense: 5′-CACTGCCTCCTAGCTTGTCC-3′

These primers are reported to be extremely specific and to not amplify genomic DNA. PCR conditions were: 50°C for 2 min, 95°C for 2 min, followed by 40 cycles of 95°C/15 s, annealing at 56°C/30 s and 72°C/30 s. All reactions were performed in triplicate using RNA from two different sets of samples.

### Mouse xenograft model and tumor growth delay experiments

Four-week-old female NOD/SCID mice (Harlan, Indianapolis, IN) were maintained under pathogen-free conditions and given food and water ad libitum. Experiments were carried out in accordance with the Catanzaro University Institutional Animal Care and Use Committee guidelines, using an approved protocol. At 6 weeks of age, mice were subcutaneously injected with 2.5 × 10^6^ ADF cells suspended in 200 µl of a 1:1 solution containing DMEM without serum plus Matrigel solution (BD Biosciences, San José, CA) in the dorsal posterior-lateral right region. Tumors were allowed to grow for four days, then mice were randomly assigned to four groups and treated with vehicle alone (DMSO, control group, six animals), SI113 (eight animals), VCR (eight animals) or SI113 plus VCR (eight animals) for five days/week. Tumors were measured every four days by caliper in two perpendicular diameters (a = smaller diameter; b = larger diameter) and the tumor volume was calculated by using the modified ellipsoid formula 1/2(Length × Width^2^). Mice, under general anesthesia, were sacrificed by vertebral dislocation.

### Statistical analysis

Unless otherwise specified, all tests were done in triplicate and experiments performed at least three times. Results are expressed as a mean ± Standard Error (SE). Differences between groups were analyzed using the One-way ANOVA test followed by the Tukey’s Multiple Comparison Test (GraphPad Prism v5). Symbols (*; ^; §) denote statistical significance as indicated in the Figure legends (one symbol <0.05; two symbols <0.01; three symbols <0.001).

In Figure 6A, differences between groups were analyzed using the Student’s two-tailed *t* test (GraphPad Prism v5). Asterisks denote statistical significance.

The assessment of synergy between two drugs was done using the algorithm described by Fransson *et al.* [24]. Synergy is characterized by a Combination Index <0.8, while a Combination Index between 0.8 and 1.2 indicates an additive effect; values >1.2 indicate an antagonistic effect.

## SUPPLEMENTARY MATERIALS FIGURES



## Abbreviations

(GBM): Glioblastoma multiforme
(RANBP1): RAN-binding protein 1
(SGK1): Serum- and glucocorticoid-regulated kinase 1
(VCR): Vincristine
(EPO-A): Epothilone A
(EPO-B): Epothilone B
(ISP): Ispinesib
(IC50): Half-maximal inhibitory concentration
(BBB): Blood-Brain Barrier
(CCNU): Lomustine
