# Supplementary material for: The small molecule SI113 synergizes with mitotic spindle poisons in arresting the growth of human glioblastoma multiforme
Source: Oncotarget. 2017 Nov 18;8(67):110743–55. doi: 10.18632/oncotarget.22500 (PMC5762281; doi:10.18632/oncotarget.22500)
Supplement: Supplementary file 1 [file oncotarget-08-110743-s001.pdf]

The small molecule SI113 synergizes with mitotic spindle poisons in arresting the growth of human glioblastoma multiforme

SUPPLEMENTARY MATERIALS

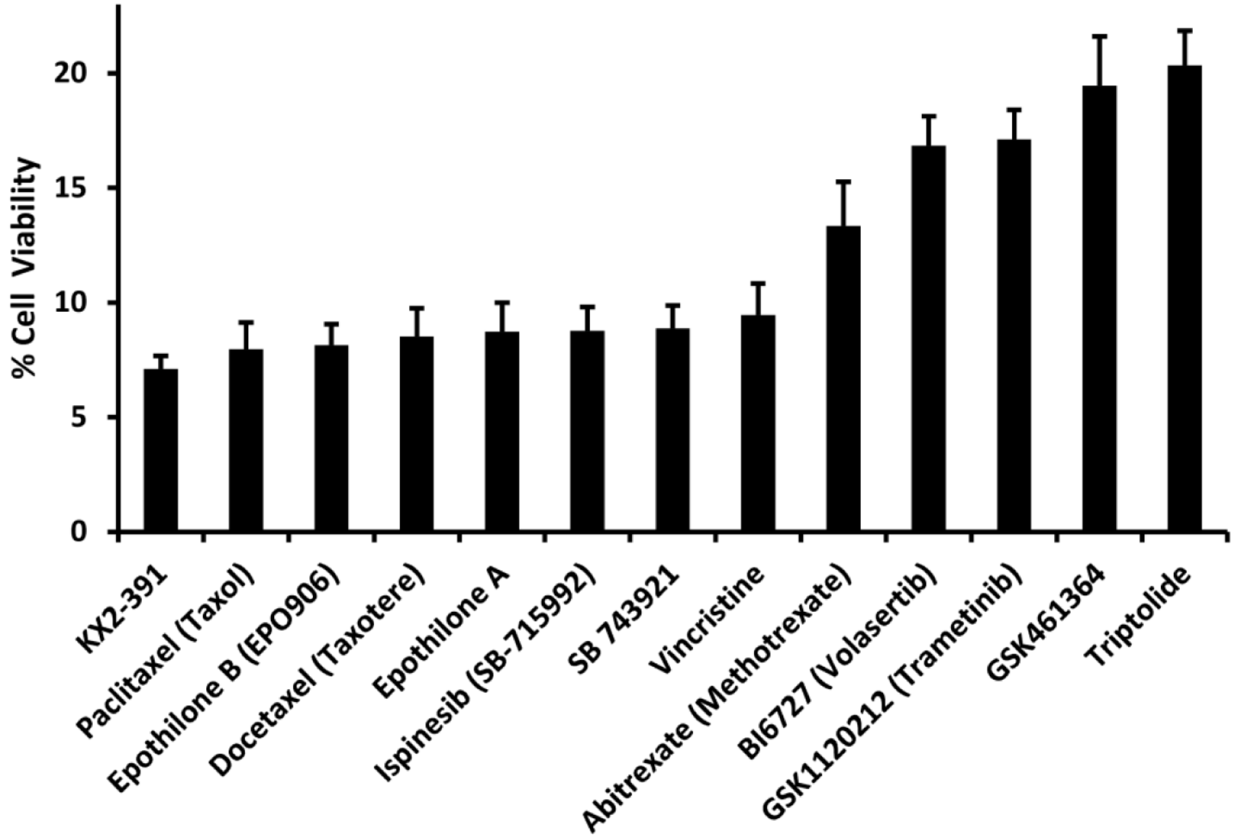

Supplementary Figure 1: Compounds able to reduce ADF cells viability. Histogram showing the effect of the library compounds able to reduce, at a 10 nM concentration for 48 h, ADF cell viability below 20%. Data for each single compound were reported as percent inhibition toward cells treated with solvent (DMSO) alone.

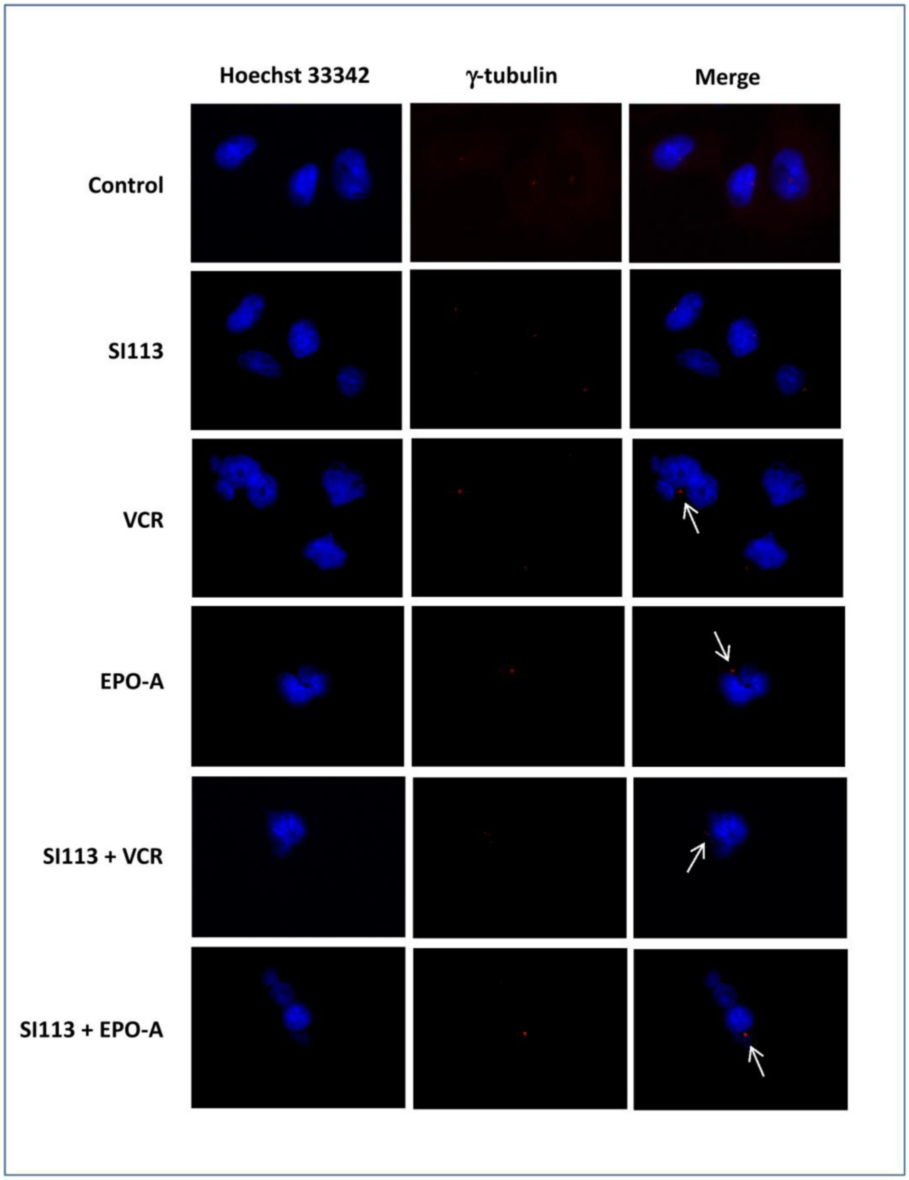

Supplementary Figure 2: Spindle poisons induce mitotic aberrations in ADF cells - Centrosome aberrations. ADF cells were treated as in Figure 3 and then stained with Hoechst 33342 to highlight nuclei (blue, left column) and γ-tubulin (fluorescent antibody, red, central column). Merging is shown in the right column. Cells incubated in the presence of VCR or EPO-A, regardless the presence or absence of SI113, display over-duplicated and clustered centrosomes (arrows).

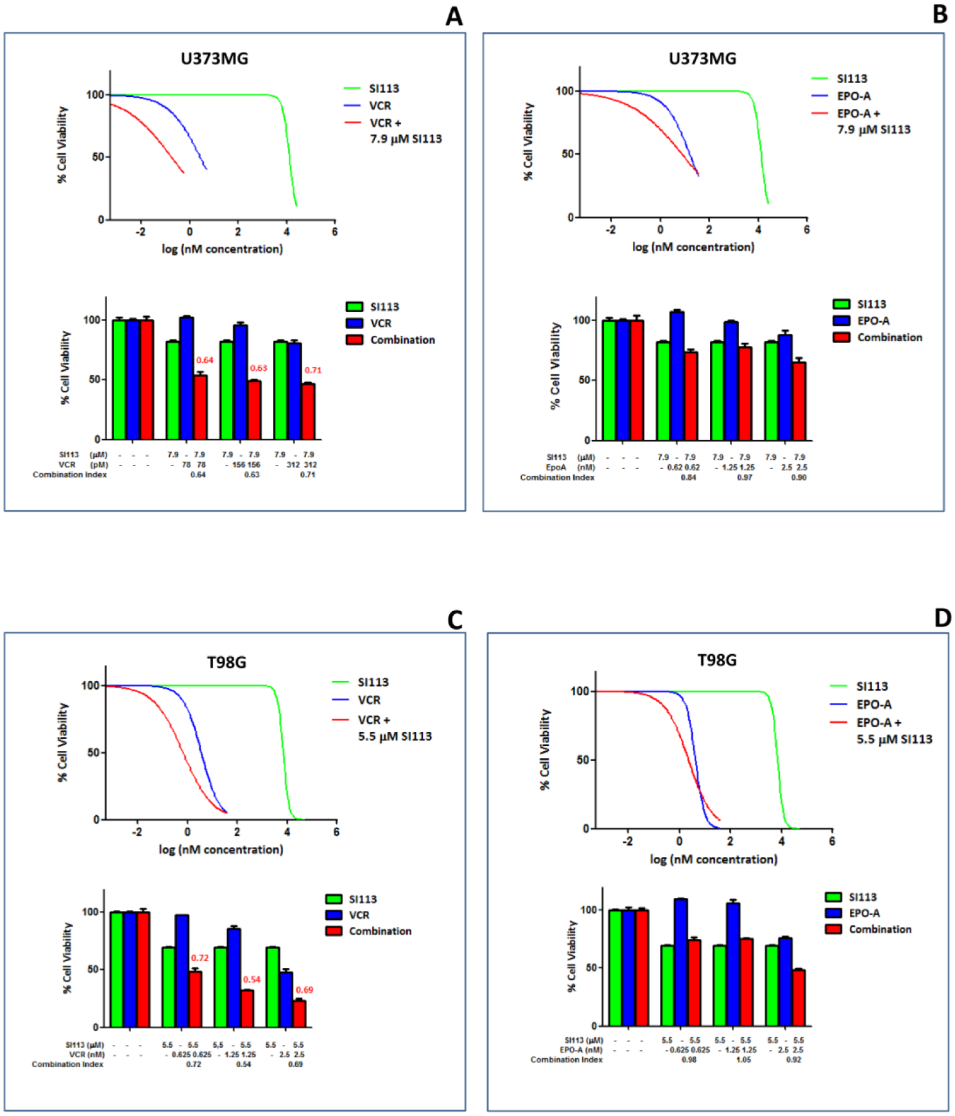

Supplementary Figure 3: Effect of SI113 plus VCR, and SI113 plus EPO-A in restraining the growth of the U373MG and T98G cells – cell viability assay. (**A**) Dose-response curves showing the effect of SI113 (green), VCR (blue) and VCR plus a constant 7.9 µM SI113 concentration (red) on percent viability of U373MG cells. Histograms show U373MG cell viability at selected drug concentrations, as indicated, to highlight the effect of the association of the two drugs. Synergy is characterized by a Combination Index <0.8 and its value is reported in red. (**B**) As in panel A, except for the use of EPO-A as the companion drug. (**C**) Dose-response curves showing the effect of SI113 (green), VCR (blue) and VCR plus a constant 5.5 µM SI113 concentration (red) on percent viability of T98G cells. Histograms show T98G cell viability at selected drug concentrations, as indicated, to highlight the effect of the association of the two drugs. Synergy is characterized by a Combination Index <0.8 and its value is reported in red. (**D**) As in panel C, except for the use of EPO-A as the companion drug. Control values were generated by adding the maximum amount of solvent(s) to the cells.

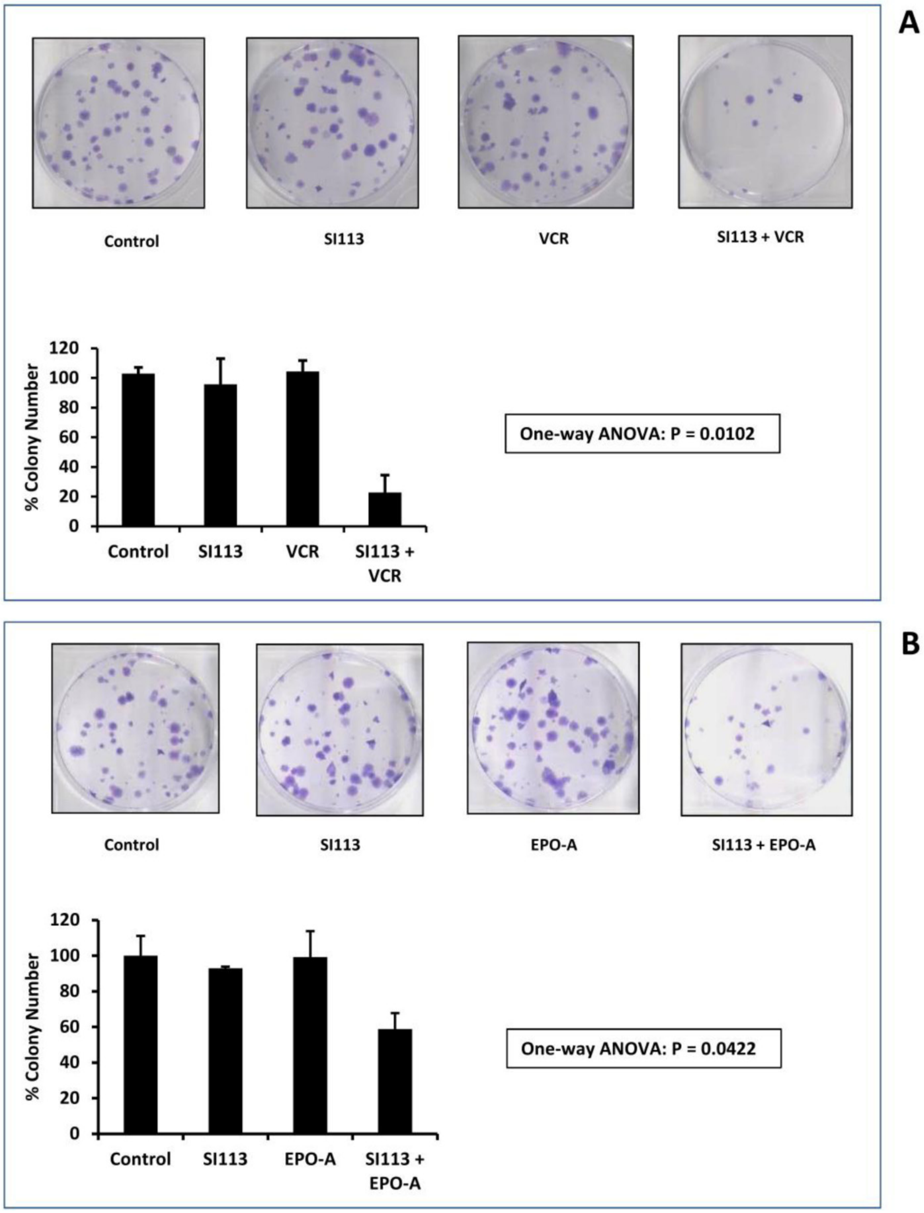

Supplementary Figure 4: Effect of SI113 plus VCR, and SI113 plus EPO-A in restraining the growth of the U373MG cells – clonogenic assay. (**A**) U373MG cells were exposed to solvent(s) (Control), 7.9 µM SI113, 78 pM VCR, or their association, for 48 h and then allowed to grow and form colonies for the subsequent 12 d. Cell colonies, after staining with crystal violet, were counted and the values reported as percent colony number in the histogram. (**B**) As in panel A, except for the use of 0.8 nM EPO-A as the companion drug. Statistical significance is also indicated

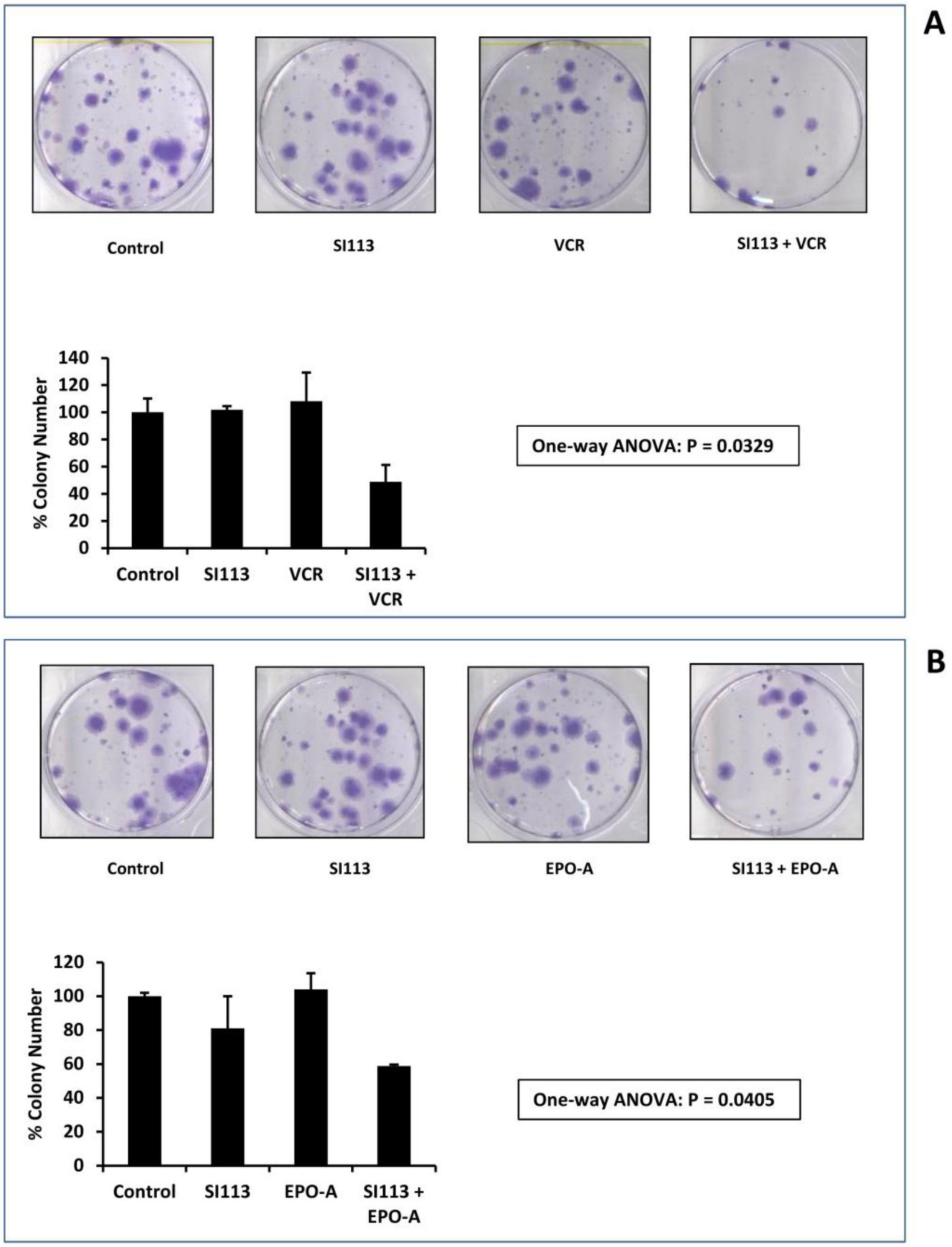

Supplementary Figure 5: Effect of SI113 plus VCR, and SI113 plus EPO-A in restraining the growth of the T98G cells – clonogenic assay. (**A**) T98G cells were exposed to solvent(s) (Control), 3 µM SI113, 0.8 nM VCR, or their association, for 48 h and then allowed to grow and form colonies for the subsequent 12 d. Cell colonies, after staining with crystal violet, were counted and the values reported as percent colony number in the histogram. (**B**) As in panel A, except for the use of 0.8 nM EPO-A as the companion drug. Statistical significance is also indicated.

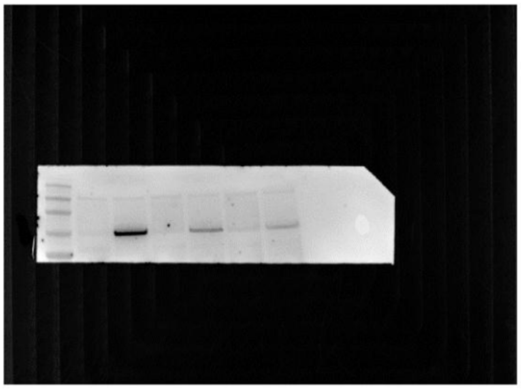

Cleaved PARP

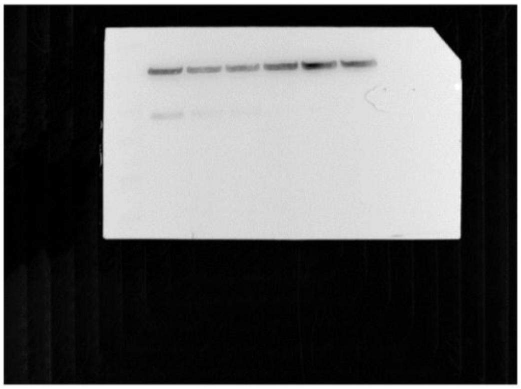

β-actin

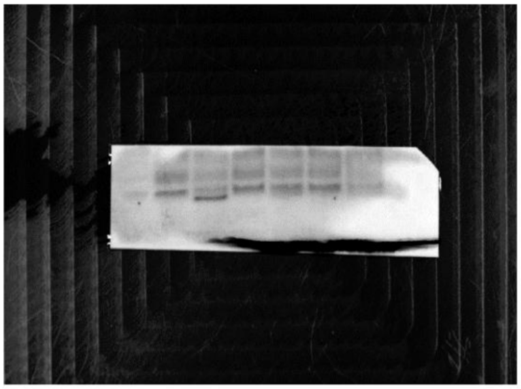

LC3 I/II

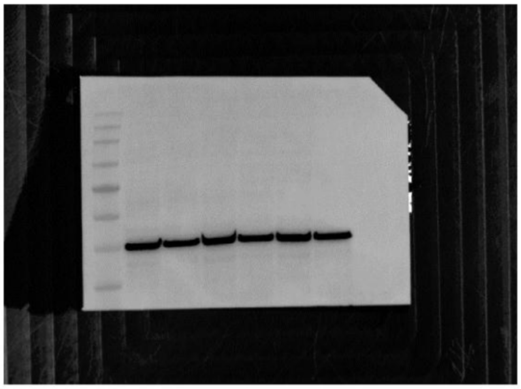

β-actin

Supplementary Figure 6: Uncut Western blot images. Original blots that have been cropped to obtain the images in Figure 2.
